# Secondary Holocord Syringomyelia Associated With Spinal Hemangioblastoma in a 29-Year-Old Female

**DOI:** 10.7759/cureus.40022

**Published:** 2023-06-06

**Authors:** Eric Chun-Pu Chu, Edouard Sabourdy, Benjamin Cheong

**Affiliations:** 1 Chiropractic and Physiotherapy Centre, New York Medical Group, Hong Kong, CHN; 2 Chiropractic Clinic, FV Hospital, Ho Chi Minh City, VNM

**Keywords:** chiropractor, chiropractic, spinal tumor, hemangioblastoma, syringomyelia

## Abstract

Syringomyelia is a neurological condition characterized by the presence of fluid-filled cavities within the spinal cord, resulting in progressive neurological deficits. Secondary holocord syringomyelia, a rare manifestation in the entire spinal cord, is associated with spinal hemangioblastomas. We report the case of a 29-year-old female who presented with neck and bilateral upper limb pain and numbness. She was diagnosed with secondary holocord syringomyelia associated with a spinal hemangioblastoma and underwent conservative management. Magnetic resonance imaging plays a critical role in diagnosing neurological conditions. The management of spinal hemangioblastomas and syringomyelia can be challenging and requires a multidisciplinary approach to patient care. In this report, we discuss the clinical presentation, diagnosis, and management of a patient with secondary holocord syringomyelia associated with spinal hemangioblastoma.

## Introduction

Spinal hemangioblastomas are rare tumors that account for 1%-2.5% of central nervous system tumors and 1.6%-5.8% of spinal cord tumors [1]. Furthermore, they are benign and highly vascular and can compress or obstruct cerebrospinal fluid (CSF) pathways in the spinal cord [2]. They frequently cause syringomyelia and can result in a variety of neurological disorders, including physical activity disorders, paresthesia, and bowel and urinary pathologies [1]. Syringomyelia is a neurological condition characterized by the formation of fluid-filled cavities (syrinxes) within the spinal cord that can lead to progressive neurological deficits and disability [3]. The pathogenesis of syringomyelia remains incompletely understood; however, it is often associated with conditions that disrupt CSF circulation, such as Chiari malformations, arachnoiditis, and spinal tumors [3].

Secondary holocord syringomyelia, in which the entire length of the spinal cord is affected, is an even rarer manifestation of this condition, with few cases reported in the literature [4]. Early diagnosis and appropriate management of patients with spinal hemangioblastomas and associated syringomyelia are crucial for preventing permanent neurological damage and improving patient outcomes [5]. However, the clinical presentation of these patients may be nonspecific and mimic other common pathological conditions, posing a diagnostic challenge for clinicians.

Chiropractors are educated to identify red flags in clinical presentations and physical examinations that may point to a severe condition, wherein they should recommend immediate diagnostic imaging [6,7]. In particular, diagnostic imaging interpretation is a part of chiropractic education that focuses on spinal pathology [8,9]. Given the paucity of research on this subject, we present the case of a 29-year-old female with secondary holocord syringomyelia associated with spinal hemangioblastoma who initially presented with neck pain and bilateral upper limb pain with numbness. Through a comprehensive evaluation and multidisciplinary approach, the patient was diagnosed with spinal hemangioblastoma and underwent conservative management. The purpose of this case report is to highlight the importance of considering serious underlying causes in patients presenting with atypical symptoms and neurological deficits and emphasize the role of early diagnosis and intervention in improving patient outcomes.

## Case presentation

**Figure 1:**
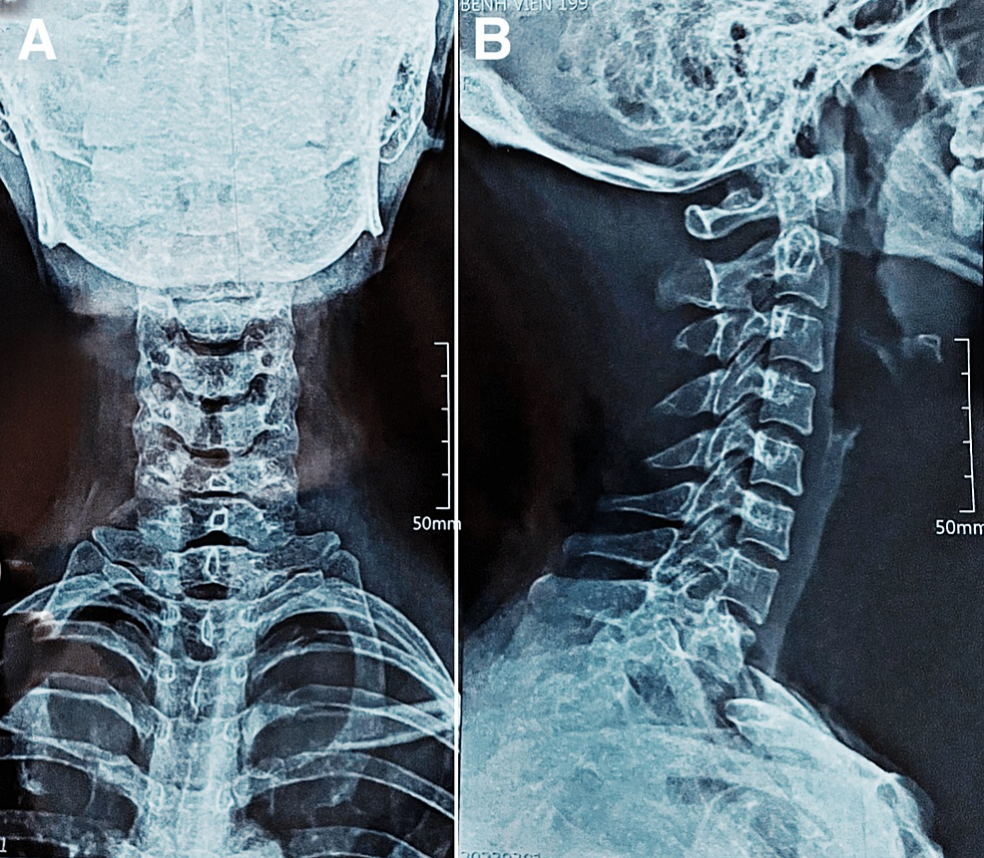
Initial cervical radiographs with unremarkable frontal (A) and sagittal (B) views.

A 29-year-old female presented with a one-month history of neck pain and two weeks of both bilateral upper limb pain and numbness affecting the fingers. The pain was described as a dull ache localized in the posterior neck and radiating to the elbows. No history of trauma was present and initial cervical radiographs were negative (Figure 1). Treatment with muscle relaxants and nonsteroidal anti-inflammatory drugs (NSAIDs) provided little relief. Additionally, the patient underwent two weeks of physiotherapy, including deep muscle massage, heat therapy, and acupuncture, without significant improvement.

However, the patient had a medical history of thyroid cancer, had undergone tumor resection two years prior, and was on thyroid hormone therapy and iron supplementation. No family history of similar illnesses was reported.

Physical examination revealed that the patient was underweight (150 cm, 38 kg) with no signs of cerebellar lesions. However, the neck’s range of motion was restricted in extension and forward flexion, with tenderness over the posterior neck. Neck swelling or lymphadenopathy was not observed. Neurological examination revealed bilateral pain sensation over the C6, C7, and C8 dermatomes, which was more pronounced in the left upper arm, and diminished sensation over the hands and fingers. Deep tendon reflexes were diminished in the right biceps, brachioradialis, and triceps, with an absent left S1 reflex. Weak grip strength was noted (10 kg right/6 kg left); however, normal power was present in both the upper and lower limbs. Finally, muscular palpation revealed tenderness over the suboccipital and trapezius muscles without deformity or swelling.

**Figure 2:**
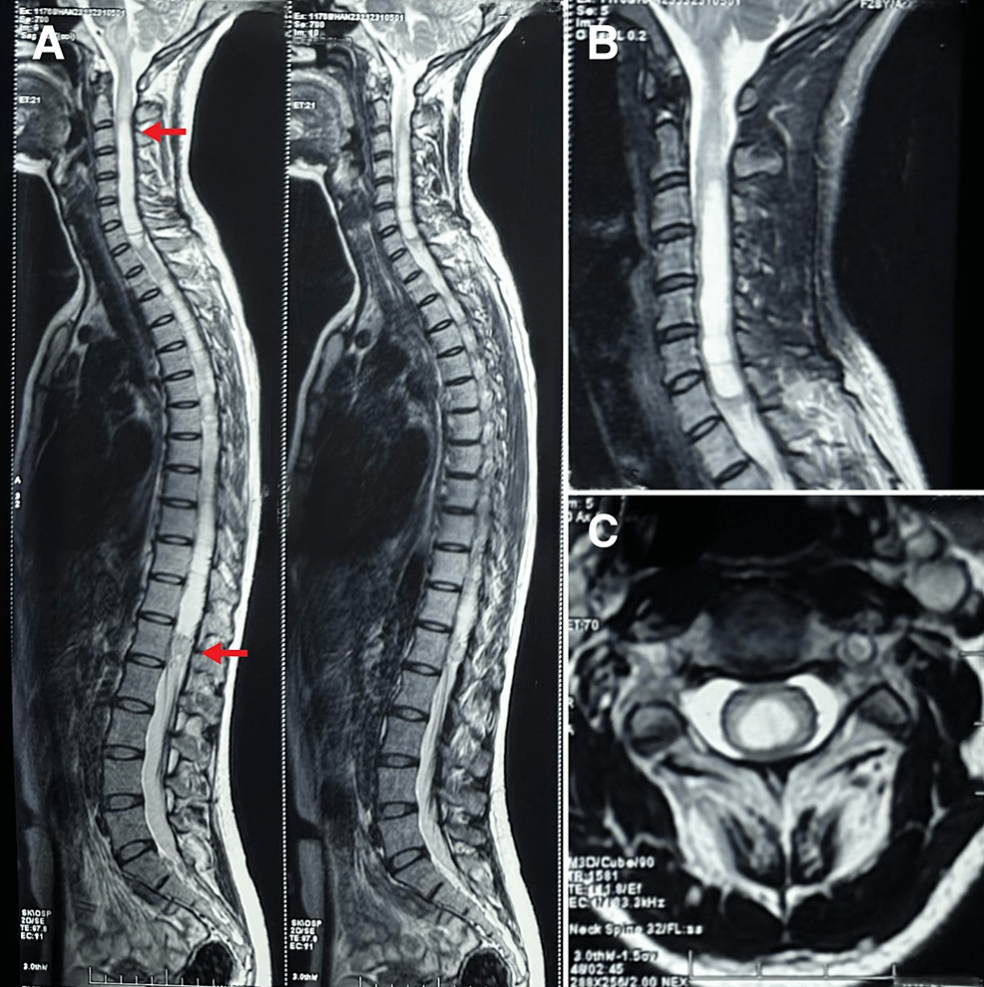
Magnetic resonance imaging (MRI). (A) A holocord syringomyelia from C2 to L1 (red arrows) and edema of the medulla and cervical cord above the syringomyelia. (B) Syringomyelia is observable at the sagittal view of the cervical spine. (C) A section showing hyperintense fluid in the spinal cord at the level of C3, which is consistent with a syrinx.

The presence of red flags, such as diminished and absent reflexes, led the chiropractor to consider differential diagnoses, including spinal cord tumor, Chiari malformation, arachnoiditis, severe cervical spondylosis, and disc herniation. Cervical and thoracic magnetic resonance imaging (MRI) was performed immediately, which revealed long syringomyelia from C2 to L1 and edema of the medulla and cervical cord above the syringomyelia (Figure 2). The chiropractor and onsite radiologist provided a working diagnosis of secondary holocord syringomyelia associated with spinal hemangioblastoma and referred the patient to the district hospital’s multidisciplinary team, which included neurosurgeons and oncologists.

Owing to the vascular nature of hemangioblastomas, performing a biopsy and surgery was deemed too risky, as it could potentially result in significant bleeding. The surgical team at the district hospital decided to provide conservative management. The conservative management plan for the patient included a combination of muscle relaxants, NSAIDs, and passive physical therapy to address the neck pain and bilateral upper limb pain and numbness. The muscle relaxant prescribed was cyclobenzaprine at a dosage of 5 mg three times daily as needed for muscle spasms and pain relief. The NSAID prescribed was ibuprofen at a dosage of 400 mg every six to eight hours as needed for inflammation and additional analgesic effects. Passive physical therapy was implemented to improve the patient’s range of motion and alleviate pain without causing further damage to the spinal cord. The therapy program consisted of gentle, controlled movements and stretches of the neck, performed by a physical therapist. The patient was advised to avoid any strenuous or weight-bearing activities that could exacerbate her symptoms or cause additional harm to the spinal cord. The therapy sessions were scheduled for multiple times per week, with the frequency and intensity adjusted based on the patient’s progress and tolerance.

Furthermore, the patient was closely monitored for any changes in her neurological status, including worsening pain, numbness, or weakness. Regular follow-up calls with the chiropractor and multidisciplinary team were scheduled to assess the patient’s response to the conservative management plan and to make any necessary adjustments. The patient was also encouraged to maintain open communication with her healthcare providers to ensure the best possible care and outcomes.

## Discussion

This case highlights the significance of vigilance and a thorough diagnostic approach in patients presenting with atypical symptoms and neurological deficits. Careful evaluation and management of syringomyelia and associated spinal hemangioblastomas are crucial for preventing permanent neurological damage and improving patient outcomes [10]. One of the key lessons learned from this case is the importance of considering serious underlying causes in patients presenting with atypical symptoms and neurological deficits. The patient’s initial presentation of neck pain and bilateral upper limb pain with numbness could have been misattributed to more common conditions such as cervical radiculopathy or myofascial pain syndrome [11]. However, the persistence of her symptoms and the presence of additional neurological deficits warranted further investigation, ultimately leading to the correct diagnosis.

Imaging studies, particularly MRI, play a critical role in the diagnosis of patients with suspected syringomyelia and spinal hemangioblastomas [11]. Patients with spinal tumors [12-15] and metastasis [16-19] have been constantly diagnosed at chiropractic clinics by MRI findings. In this case, cervical and thoracic MRI revealed the presence of spinal hemangioblastoma and associated holocord syringomyelia, allowing appropriate referral and management. When evaluating patients with symptoms suggestive of spinal cord pathology, clinicians must be prepared to order appropriate imaging studies as early diagnosis can significantly affect the patient’s prognosis [5].

Management of spinal hemangioblastomas and syringomyelia can be challenging because surgical intervention is often necessary, and the risks associated with these procedures must be carefully weighed against the potential benefits. Although surgical resection was successful in relieving symptoms [20], it is believed that the improvements may be related to changes in blood flow and not reductions in the syrinx following surgery [20]. However, treatments that directly affect the CSF flow have been reported to correct energetic and structural dysfunction in the central nervous system [21], and chiropractic management has been reported to be beneficial for neck pain associated with syringomyelia after spinal surgery [22]. In this case, the patient underwent conservative management of the spinal hemangioblastoma. This case highlights the need for a multidisciplinary approach to patient care, with chiropractors being vigilant in identifying atypical presentations and referring patients for further evaluation and management.

## Conclusions

This case report emphasizes the importance of early recognition and prompt referral of patients with atypical symptoms and neurological deficits, as evidenced by the diagnosis of secondary holocord syringomyelia associated with a spinal hemangioblastoma in a 29-year-old female. The case highlights the critical role of imaging, particularly MRI, in the diagnostic process and the need for a multidisciplinary approach to patient care. Clinicians, including chiropractors, should maintain a high index of suspicion for serious underlying conditions and collaborate with other healthcare professionals for appropriate evaluation and management to improve patient outcomes.
